# Efficacy and safety of umbilical cord-derived mesenchymal stem cells in Chinese adults with type 2 diabetes: a single-center, double-blinded, randomized, placebo-controlled phase II trial

**DOI:** 10.1186/s13287-022-02848-6

**Published:** 2022-05-03

**Authors:** Li Zang, Yijun Li, Haojie Hao, Jiejie Liu, Yu Cheng, Bing Li, Yaqi Yin, Qian Zhang, Fei Gao, Haibin Wang, Shi Gu, Jia Li, Fengxiang Lin, Yingfei Zhu, Guanglei Tian, Yulong Chen, Weijun Gu, Jin Du, Kang Chen, Qinghua Guo, Guoqing Yang, Yu Pei, Wenhua Yan, Xianling Wang, Junhua Meng, Saichun Zhang, Jianming Ba, Zhaohui Lyu, Jingtao Dou, Weidong Han, Yiming Mu

**Affiliations:** 1 Department of Endocrinology, The First Medical Center of Chinese PLA General Hospital, No. 28 Fuxing Road, Beijing, 100853 China; 2 Department of Biotherapy, The First Medical Center of Chinese PLA General Hospital, No. 28 Fuxing Road, Beijing, 100853 China

**Keywords:** Type 2 diabetes mellitus, Umbilical cord-derived mesenchymal stem cells, Glycated hemoglobin A1c, Islet β-cell function, Insulin resistance

## Abstract

**Background:**

To determine the efficacy and safety of umbilical cord-derived mesenchymal stem cells (UC-MSCs) in Chinese adults with type 2 diabetes mellitus (T2DM).

**Methods:**

In this single-center, double-blinded, randomized, placebo-controlled phase II trial, 91 patients were randomly assigned to receive intravenous infusion of UC-MSCs (*n* = 45) or placebo (*n* = 46) three times with 4-week intervals and followed up for 48 weeks from October 2015 to December 2018. The primary endpoint was the percentage of patients with glycated hemoglobin (HbA1c) levels of < 7.0% and daily insulin reduction of ≥ 50% at 48 weeks. Additional endpoints were changes of metabolic control, islet β-cell function, insulin resistance, and safety.

**Results:**

At 48 weeks, 20% of the patients in the UC-MSCs group and 4.55% in the placebo group reached the primary endpoint (*p* < 0.05, 95% confidence interval (CI) 2.25–28.66%). The percentage of insulin reduction of the UC-MSCs group was significantly higher than that of the placebo group (27.78% versus 15.62%, *p* < 0.05). The levels of HbA1c decreased 1.31% (9.02 ± 1.27% to 7.52 ± 1.07%, *p* < 0.01) in the UC-MSCs group, and only 0.63% in the placebo group (8.89 ± 1.11% to 8.19 ± 1.02%, *p*˃0.05; *p* = 0.0081 between both groups). The glucose infusion rate (GIR) increased significantly in the UC-MSCs group (from 3.12 to 4.76 mg/min/kg, *p* < 0.01), whereas no significant change was observed in the placebo group (from 3.26 to 3.60 mg/min/kg, *p* ˃ 0.05; *p* < 0.01 between both groups). There was no improvement in islet β-cell function in both groups. No major UC-MSCs transplantation-related adverse events occurred.

**Conclusions:**

UC-MSCs transplantation could be a potential therapeutic approach for Chinese adults with T2DM.

*Trial registration* This study was registered on ClinicalTrials.gov (identifier: NCT02302599).

**Supplementary Information:**

The online version contains supplementary material available at 10.1186/s13287-022-02848-6.

## Background

Type 2 diabetes mellitus (T2DM) is a heterogeneous syndrome that is characterized by a progressive deterioration in glycemic control caused by decreased insulin sensitivity and diminished insulin secretion, which has become a serious threat to human health worldwide due to its high prevalence and severe morbidity. Diet control, physical exercise, and glucose-lowering medications have been shown to temporarily improve hyperglycemia but cannot inhibit the pathogenesis or reduce the morbidity of T2DM. Therefore, the development of more effective approaches for the treatment of T2DM is required.

Mesenchymal stem cells (MSCs) are a type of adult stem cells with profound anti-inflammatory and immunomodulatory capacities by secreting a variety of cytokines and immunosuppressive molecules [1, 2]. They have been successfully applied in the treatment of different types of autoimmune diseases, such as stroke [3], myocardial infarction [4], rheumatoid arthritis, systemic lupus erythematosus [5, 6], and graft versus host disease [7]. Chronic inflammatory responses and immune disorders in insulin-sensitive tissues and pancreatic islets contribute to insulin resistance, islet β-cell destruction, and the onset of T2DM. Therefore, MSCs transplantation might be a therapeutic option for T2DM. Previous animal studies have demonstrated that MSCs treatment ameliorated hyperglycemia by promoting pancreatic islet recovery and alleviating insulin resistance [8, 9]. Moreover, an increasing number of clinical trials have reported the therapeutic effects and safety of MSCs transplantation in patients with T2DM [10–20]. MSCs can be derived from various tissues (e.g., bone marrow, adipose tissues, and umbilical cord), and the procurement or use of MSCs is deemed non-controversial. MSCs were first isolated from bone marrow [21], which was considered the most accessible source of MSCs for the treatment of T2DM [22]. Several clinical trials have confirmed the therapeutic potential of bone marrow-derived MSCs (BM-MSCs) in T2DM [10, 12, 14–17]. Umbilical cord-derived MSCs (UC-MSCs) share similar immunosuppressive properties as BM-MSCs and possess clinical potential for T2DM owing to their low-cost, pain-free, high-yield, rapid-collection, and non-immunogenicity characteristics [19]. However, few clinical studies have focused on the treatment of T2DM with UC-MSCs and no clinical trials have reported the effect of UC-MSCs on insulin resistance in patients with T2DM. Therefore, a single-center, randomized, double-blinded, placebo-controlled phase II trial was performed to explore the efficacy and safety of intravenous infusion of UC-MSCs in patients with T2DM.

## Methods

### Study design and participants

This prospective, single-center, randomized, double-blinded, placebo-controlled phase II trial was performed at the First Medical Center of Chinese PLA General Hospital (PLAGH; Beijing, China) from October 2015 to December 2018. The study protocol was approved by the Ethical Committee of the First Medical Center of PLAGH (Approval No. 2013-107-01) and conformed to the Declaration of Helsinki guidelines. All participants provided written informed consent before recruitment. This study was registered on ClinicalTrials.gov (identifier: NCT02302599).

Patients who met the following inclusion criteria were qualified for enrollment: (1) aged between 20 and 65 years; (2) diagnosed with T2DM for < 20 years (HbA1c levels between 7.0% and 12.0%, inclusive; (3) inadequately controlled by stable insulin therapy (0.5–1.0 U/kg/day) with metformin for ≥ 3 months; (4) with fasting C-peptide levels of ≥ 1 ng/mL; and (5) with a body mass index (BMI) of 24–40 kg/m^2^. Patients were excluded from this study if they had ketonuria, tumors, serum creatinine levels < 175 μmol/L, previously diagnosed with myocardial infarction, current angina or heart failure, ˃ 1 major vascular event, retinopathy that required laser treatment, malignant hypertension, an uncorrected endocrine disorder, occupations that precluded insulin therapy, severe concurrent illness that limited life expectancy, inadequate understanding of the study protocol, drug abuse, planning pregnancy, and an allergic constitution. The full list of inclusion and exclusion criteria is provided in the appendix.

### Randomization and masking

The Interactive Web Response System was used to assign eligible patients into the trial in a 1:1 ratio according to age of the patients (< 40 or ≥ 40 years), BMI (< 28, ≥ 28, or ≥ 32 kg/m^2^), duration of T2DM (< 5, ≥ 5, or ≥ 10 years), and HbA1c levels (< 7.5%, ≥ 7.5% or ˃9%). All patients were assessed at baseline and pre-established follow-up time points (at 9, 20, 32, and 48 weeks). All investigators and participants were masked to treatment allocation.

### Procedures

Human umbilical cords were obtained from healthy women who gave birth in the First Medical Center of Chinese PLAGH. All subjects provided informed consent. All procedures were performed according to the Guidelines of the Ethics Committee of the First Medical Center of Chinese PLAGH. UC-MSCs were isolated from the gelatinous tissues surrounding the vein and artery and were characterized by phenotype analysis and cell differentiation assay as previously described [23, 24]. Patients who met the eligibility criteria were randomly assigned to receive intravenous infusion of UC-MSCs (100 mL) or the same volume and appearance of placebo (UC-MSCs suspension liquid composed of saline with 3% human albumin and 0.5 mL multivitamins) at the elbow joint three times with an interval of 4 weeks and then discharged after 24 h of observation without any adverse events. UC-MSCs at four passages were used in this study, and the total number of UC-MSCs for each transfusion was 1 × 10^6^/kg.

After discharge, patients were followed every 12 weeks for 48 weeks and required to perform self-monitoring of blood glucose (≥ 15 times per week that included a 5-point profile) on different days during follow-up. Insulin titration was based on peripheral blood glucose levels, with target fasting plasma glucose levels between 4.4 and 7.0 mmol/L. Patients were recommended to maintain a regular diabetic diet and healthy lifestyle during hospitalization and follow-up. If the total daily insulin dose of a patient was ≤ 0.2 U/kg at any time during the study, they discontinued the use of exogenous insulin and were given an oral anti-diabetic agent to prevent hypoglycemia. If a patient developed uncontrolled blood glucose with a total daily insulin dose of ˃1.5 U/kg, they discontinued the study, and the hypoglycemic strategy was adjusted according to the condition of the patient to prevent diabetic complications. Insulin requirement and HbA1c levels were assessed every 12 weeks. Islet β-cell function was indicated by the levels of fasting C-peptide, fold change in C-peptide levels stimulated by intravenous administration of 1 mg glucagon, and the C-peptide area under the curve (AUCC-pep) in the oral glucose tolerance test (OGTT, 6 points) at baseline and 9, 20, and 48 weeks. The AUCC-pep was calculated using the trapezoidal method. The glucose infusion rate (GIR) measured by a hyperinsulinemic-euglycemic clamp (HEC) was used to indicate insulin resistance at baseline, 9 and 48 weeks. The potential risk of UC-MSCs transplantation was observed during treatment and follow-up.

### Endpoints

The primary endpoint was the percentage of patients with HbA1c levels of < 7.0% and daily insulin reduction of ≥ 50% from baseline to 48 weeks. The secondary endpoints were changes in insulin requirement, HbA1c levels, and percentage of patients with HbA1c levels of < 7.0% from baseline to 9, 20, 32, and 48 weeks; changes in islet β-cell function (indicated by fasting C-peptide levels, glucagon-stimulated C-peptide changes, and AUCC-pep) from baseline to 9, 20, and 48 weeks; and changes of insulin resistance (indicated by GIR) from baseline to 9 and 48 weeks.

The primary safety endpoints included immediate adverse events following intravenous administration of UC-MSCs or placebo, adverse events related to hypersensitivity, injection site reactions, infection, tumor formation, abnormal vital signs in electrocardiogram, ultrasound scan, and laboratory tests, and occurrence of hypoglycemia in the safety population (defined as all participants who received ≥ 1 dose of UC-MSCs or placebo). More details and the review process are provided in the appendix.

### Statistical analysis

Data were analyzed using the SAS software (version 9.4). Normally distributed continuous variables were presented as mean ± standard deviation (SD), while non-normally distributed variables were expressed as median (IQR). An independent two-sample *t*-test was used to determine the differences of quantitative variables (e.g., fasting plasma glucose levels, HbA1c levels, fasting C-peptide levels, insulin dose, and GIR) between both groups. Non-normally distributed variables were analyzed using the Wilcoxon rank-sum test. Categorical variables were expressed as frequency and percentile. A Chi-squared (χ^2^) test or Fisher’s exact test was used to determine statistical significances between the two groups. The paired *t*-test or Wilcoxon matched-pairs signed-rank test was used for within-group comparisons. The χ^2^ test was performed to analyze the differences in the percentage of patients with HbA1c levels of < 7.0% and daily insulin reduction of ≥ 50% between both groups. The 95% confidence interval (CI) of the rate difference was also determined. A *p*-value < 0.05 was considered statistically significant.

## Results

### Characterization of human UC-MSCs

Cultured human UC-MSCs had a bipolar spindle-shaped and fibroblast-like morphology (Additional file 1: Fig. S1A). Then, the immunophenotypic features and multi-lineage differentiation potential of adherent cells were examined. As shown in Additional file 1: Fig. 1B, cells expressed surface markers of UC-MSCs, including CD90, CD73, and CD105, and negative surface markers of UC-MSCs, including CD34, CD45, and HLA-DR, were not detected. Moreover, UC-MSCs exhibited differentiation potential to osteoblasts (Additional file 1: Fig. S1C) and adipocytes (Additional file 1: Fig. S1D).

### Study population

**Fig. 1:**
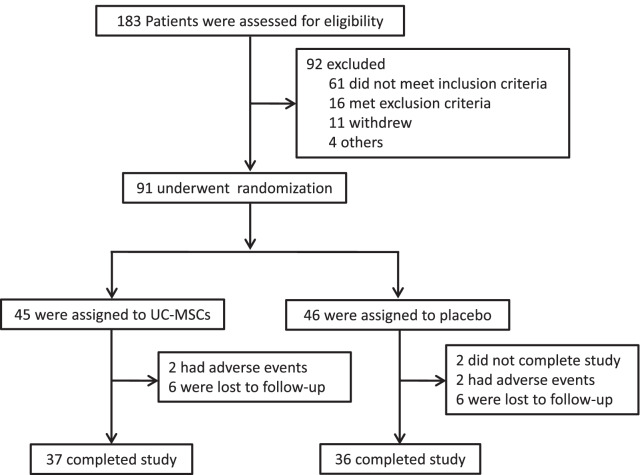
Flowchart of the study. UC-MSCs, umbilical cord mesenchymal stem cells

Between October 2015 and December 2018, 183 patients were screened for eligibility and 91 were finally recruited. Enrolled subjects were randomly assigned to receive UC-MSCs transplantation (*n* = 45) or placebo (*n* = 46). A total of 73 (86.8%) patients completed the trial, with 37 (82.2%) patients in the UC-MSCs group and 36 (78.3%) patients in the placebo group. Two patients in the placebo group did not complete all treatments. Two patients were withdrawn due to adverse events, and six patients were lost to follow-up in both groups (Fig. 1). The demographic and clinical characteristics of patients at baseline were balanced between the two groups (Table 1).

**Table 1:** Baseline characteristics of patients

Characteristics	UC-MSCS (*N* = 45)	Placebo (*N* = 46)	*p*-value
Age (years)	50.00 ± 9.38	50.45 ± 8.03	0.8068
Gender (male) (*n* (%))	28 (62.22%)	30 (68.18%)	0.5552
BMI (kg/m^2^)	28.69 ± 3.35	28.13 ± 3.04	0.4158
Duration of diabetes (years)	11.44 ± 4.78	11.70 ± 3.96	0.7808
Insulin requirement (U/day)	57.36 + 18.90	56.41 + 12.54	0.7819
Insulin requirement (U/kg/day)	0.72 ± 0.20	0.71 ± 0.15	0.6992
Fasting plasma glucose (mmol/L)	8.71 ± 2.16	8.58 ± 1.93	0.7737
Glycated hemoglobin A1c (%)	9.02 ± 1.27	8.89 ± 1.11	0.6173
Fasting C-peptide (ng/mL)	2.01 ± 0.70	1.93 ± 0.65	0.5905
GIR (mg/min/kg)	3.12 ± 1.46	3.26 ± 1.16	0.6177

Data are shown as mean ± SD or n (%). GIR, Glucose infusion rate

### Efficacy

Compared with the placebo group, more patients in the UC-MSCs group achieved the primary endpoint. At 48 weeks following treatment, 20% of the patients in the UC-MSCs group achieved the goal of HbA1c < 7.0% and daily insulin reduction ≥ 50%, compared with 4.55% in the placebo group (*p* < 0.05, Table 2). The 95% CI for rate difference between both groups was 2.25–28.66%.

**Table 2:** The percentage of patients with HbA1c levels of < 7.0% and daily insulin reduction of ≥ 50% at 9–48 weeks of follow-up

	UC-MSCS (% of patients)	PLACEBO (% of patients)	*p*-value
9 weeks	8.89	2.27	0.3607
20 weeks	15.56	9.09	0.3542
32 weeks	15.56	9.09	0.3542
48 weeks	20.00	4.55	0.0268

**Fig. 2:**
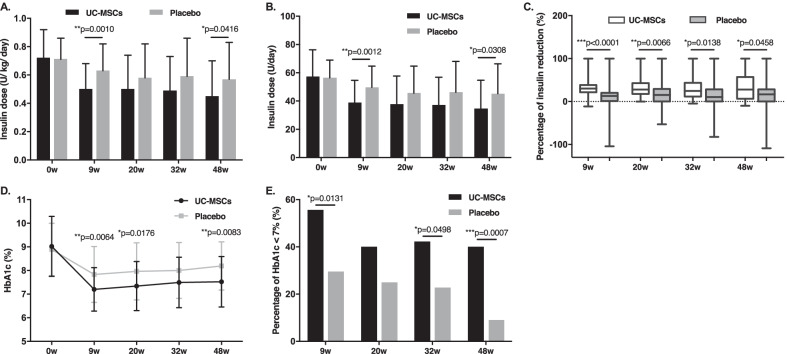
Insulin requirement and HbA1c levels after treatment with UC-MSCs or placebo. *p < 0.05, **p < 0.01, ***p < 0.001 between both groups. HbA1c, glycated hemoglobin A1c

In both groups, the daily insulin requirement at 9, 20, 32, and 48 weeks progressively decreased compared with their respective baseline values (*p* < 0.01 versus baseline for both groups). However, insulin requirement of patients in the UC-MSCs group was still lower than that of the placebo group at 9, 20, 32, and 48 weeks (0.50 ± 0.18 versus 0.63 ± 0.19 U/kg/day, *p* < 0.01; 0.50 ± 0.24 versus 0.58 ± 0.24 U/kg/day, *p*˃0.05; 0.49 ± 0.24 versus 0.59 ± 0.27 U/kg/day, *p˃*0.05; 0.45 ± 0.25 versus 0.57 ± 0.26 U/kg/day, *p* < 0.05, respectively) (Fig. 2A). Similar patterns were observed in total insulin dose of the two groups at the same time points (38.80 ± 15.89 versus 49.75 ± 14.92 U/day, *p* < 0.01; 37.81 ± 19.87 versus 45.56 ± 19.14 U/day, *p*˃0.05; 37.32 ± 19.63 versus 46.19 ± 21.85 U/day, *p*˃0.05; 34.51 ± 20.19 versus 45.19 ± 21.21 U/day, *p* < 0.05, respectively) (Fig. 2B). The percentage of insulin reduction in the UC-MSCs group was significantly higher than that of the placebo group at 9, 20, 32, and 48 weeks (30.00% versus 12.77%, *p* < 0.01; 27.83% versus 15.25%, *p* < 0.01; 24.44% versus 10.24%, *p* < 0.05, 27.78% versus 15.62%, *p* < 0.05, respectively) (Fig. 2C). Overall, 13.5% (5/37) patients became insulin-free at 8–24 weeks (12 ± 7.6 weeks) after UC-MSCs transplantation and remained insulin-free without re-use for 37.2 ± 15.2 weeks. No patient in the placebo group became insulin-free.

The HbA1c levels declined after UC-MSCs transplantation with a maximum decrease observed at 9 weeks after treatment, and slightly increased at 20, 32, and 48 weeks of follow-up; however, they were still lower than the values at baseline (9.02 ± 1.27%, 7.20 ± 0.92%, 7.34 ± 1.04%, 7.49 ± 1.07%, and 7.52 ± 1.07% at baseline and 9, 20, 32, and 48 weeks, respectively; *p* < 0.01 compared with baseline). In addition, the HbA1c levels of the placebo group underwent a slight decrease at 9 weeks and then gradually increased to relatively high levels (8.89 ± 1.11%, 7.83 ± 1.18%, 7.96 ± 1.21%, 8.00 ± 1.18%, and 8.19 ± 1.02% at baseline and 9, 20, 32, and 48 weeks, respectively; *p* < 0.01 compared with baseline). The HbA1c levels in the UC-MSCs group were lower than those of the placebo group at the same time points during follow-up (*p* < 0.01 at 9 and 48 weeks, *p* < 0.05 at 20 weeks, *p*˃0.05 at 32 weeks between both groups) (Fig. 2D). The HbA1c levels decreased 1.31% in the UC-MSCs group but only 0.63% in the control group at 48 weeks (*p* = 0.0081). More participants in the UC-MSCs achieved the target HbA1c levels (< 7.0%), compared with the placebo group at 9, 20, 32, and 48 weeks (55.56% versus 29.55%, *p* < 0.05; 40.00% versus 25.00%, *p*˃0.05; 42.22% versus 22.73%, *p* < 0.05; and 40.00% versus 9.09%, *p* < 0.01, respectively) (Fig. 2E). These results suggested the therapeutic potential of UC-MSCs transplantation in T2DM.

**Fig. 3:**
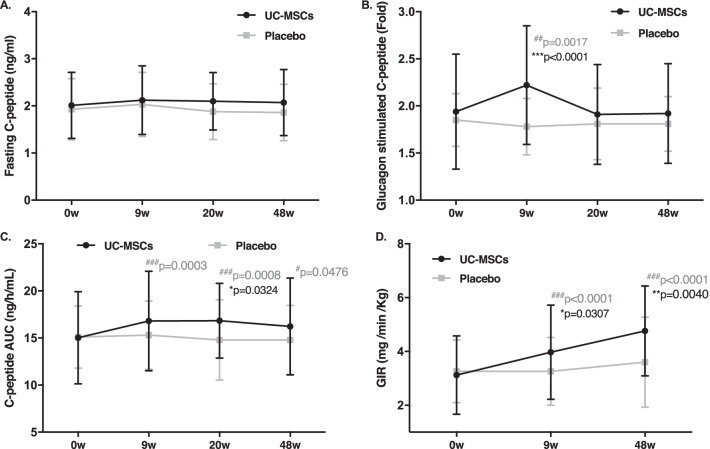
Islet β-cell function and insulin resistance after treatment with UC-MSCs or placebo. *p < 0.05, **p < 0.01, ***p < 0.001 between both groups; ^#^*p* < 0.05; ^##^ *p* < 0.01; ^###^*p* < 0.001 versus baseline values. GIR, glucose infusion rate

The fasting C-peptide levels in the UC-MSCs group did not significantly change after treatment (2.01 ± 0.70, 2.12 ± 0.73, 2.10 ± 0.61, and 2.07 ± 0.70 ng/mL at baseline and 9, 20, and 48 weeks, respectively; *p*˃0.05 compared with baseline), but decreased in the placebo group (1.93 ± 0.65, 1.93 ± 0.68, 1.88 ± 0.59, and 1.86 ± 0.60 ng/mL at baseline and 9, 20, and 48 weeks, respectively; *p*˃0.05) (*p*˃0.05 between both groups) (Fig. 3A). The glucagon-stimulated fold change in C-peptide levels in the UC-MSCs group increased at 9 weeks and progressively decreased at 20 and 48 weeks (1.94 ± 0.61 ng/mL, 2.22 ± 0.63 ng/mL, 1.91 ± 0.53 ng/mL, 1.92 ± 0.53 ng/mL at baseline and 9, 20, and 48 weeks, respectively; *p* < 0.01 at 9 weeks, *p* ˃ 0.05 at 20 and 48 weeks compared with baseline). The fold change in C-peptide levels gradually decreased in the placebo group (1.85 ± 0.28, 1.78 ± 0.30, 1.81 ± 0.38, and 1.8 1 ± 0.29 ng/mL at baseline and 9, 20, and 48 weeks, respectively) (*p* < 0.05 at 9 weeks, *p* ˃ 0.05 at 20 and 48 weeks between both groups) (Fig. 3B). The AUCC-pep in the UC-MSCs group increased at 9 and 20 weeks and slightly decreased at 48 weeks (15.03 ± 4.9, 16.80 ± 5.29, 16.84 ± 3.97, and 16.23 ± 5.14 ng/h/mL at baseline and 9, 20, and 48 weeks, respectively; *p* < 0.01 at 9 and 20 weeks, *p* < 0.05 at 48 weeks compared with baseline), and in the placebo group gradually decreased (15.10 ± 3.30, 15.31 ± 3.63, 14.79 ± 4.27, and 14.79 ± 3.67 ng/h/mL at baseline and 9, 20, and 48 weeks, respectively, *p*˃0.05) (*p* < 0.05 at 20 weeks between both groups) (Fig. 3C). These results indicated that UC-MSCs did not significantly improve islet β-cell function in patients with T2DM.

The HEC results showed that UC-MSCs transplantation effectively reduced insulin resistance in patients with T2DM. The GIRs of the placebo group were 3.26, 3.26, and 3.60 mg/min/kg at baseline and 9 and 48 weeks, respectively (*p* ˃ 0.05 compared with baseline), and those of the UC-MSCs group were 3.12, 3.97, and 4.76 mg/min/kg, respectively (*p* < 0.01 at 9 and 48 weeks compared with baseline) (*p* < 0.05 at 9 weeks, *p* < 0.01 at 48 weeks between both groups) (Fig. 3D).

### Safety

All adverse events are summarized in Table 3. Two patients in the UC-MSCs group were withdrawn from the trial. One female patient had a history of hypertension and hyperlipidemia for 10 years and suffered from cerebral infarction one month after the third UC-MSCs infusion. She recovered two months after the treatment. One male patient suffered from femoral neck fracture caused by an accident 3 months after the third UC-MSCs infusion. After discussion with an independent adjudication committee, these events were not considered to be related to UC-MSCs treatment.

**Table 3:** Summary of adverse events

Treatment emergent adverse events	UC-MSCS (*N* = 45)	Placebo (*N* = 46)
Cerebral infarction	1 (2.22%)	0
Femoral neck fracture caused by accident	1 (2.22%)	0
Positive IAA	0	1 (2.27%)
Hyperplastic anemia	0	1 (2.27%)
Papillary thyroid carcinoma	0	1 (2.27%)
Prostate cancer	0	1 (2.27%)

In the placebo group, one patient with positive insulin autoantibody (IAA) and one patient with hyperplastic anemia were withdrawn from the trial. One patient was diagnosed with papillary thyroid carcinoma and one patient was diagnosed with prostate cancer on the final visit; therefore, they discontinued the study. No treatment-associated deaths were reported during the study. Other patients showed no positive tumor markers, and no tumor was detected by imaging examinations. Physical examinations and laboratory tests revealed no other UC-MSCs-related side effects within 48 weeks of follow-up. All patients (*n* = 91) remain under follow-up for potential late-onset side effects.

## Discussion

This prospective, single-center, randomized, double-blinded, placebo-controlled phase II trial showed that intravenous infusion of UC-MSCs at the elbow joint resulted in a higher percentage of patients achieving the target HbA1c levels < 7.0% and daily insulin reduction of ≥ 50% at 48 weeks compared with the placebo group. UC-MSCs treatment reduced daily insulin requirement, decreased HbA1c levels, and ameliorated insulin resistance in a time-dependent manner. No major UC-MSCs transplantation-related adverse events occurred. These results indicated the potential efficacy and safety of UC-MSCs transplantation for the treatment of T2DM.

In 2009, Bhansali et al*.* first showed that BM-MSCs transplantation significantly reduced insulin requirement and improved stimulated C-peptide levels in 10 patients with T2DM [10]. Then, a number of studies have confirmed the efficacy and safety of BM-MSCs and placenta-derived MSCs in the treatment of T2DM [11–17], including three prospective, randomized, single-blinded placebo-controlled studies [14, 15, 17]. UC-MSCs are immune-privileged, immune-suppressive cells that are readily available with few ethical issues [25]. Therefore, they might be a novel source of cell therapy for diabetes. Three studies used UC-MSCs transplantation for the treatment of T2DM in humans. One study showed that combined intravenous and intrapancreatic endovascular injection of UC-MSCs with a 5 day interval decreased HbA1c levels and insulin dose in patients with T2DM at 6 months post-treatment (41% of the patients became insulin-independent; 29% of the patients showed reduced insulin requirement by ≥ 50%). However, these indices deteriorated in the following 3–6 months [18]. In another study, 18 patients with T2DM received intravenous transfusion of UC-MSCs three times with 2-week intervals for 6 months. The results showed that 8 out of 18 patients were responsive to the treatment, manifested by reduced fasting and postprandial blood glucose levels; however, no significant difference was observed in insulin dose before and after treatment [19]. In another trial, six patients with T2DM were treated with intravenous transfusion of UC-MSCs two times with 2-week intervals and followed for ≥ 24 months. Three out of six patients became insulin-free between 25 and 43 months. The remaining patients continued to require insulin injections, although with a reduced dose [20]. These studies preliminarily confirmed the efficacy of UC-MSCs for the treatment of T2DM. However, the results of insulin requirement following treatment were not consistent. In this double-blinded, randomized, placebo-controlled study, more patients in the UC-MSCs group achieved the primary endpoint at 48 weeks following treatment compared with the placebo group, and insulin requirement decreased following UC-MSCs treatment in a time-dependent manner, which suggested the efficacy of UC-MSCs treatment for T2DM. However, the percentage of insulin reduction and the proportion of patients with insulin-free in our UC-MSCs group was lower than those reported in other studies, which might be explained by the heterogeneity of enrolled subjects.

Previous studies showed that the HbA1c levels of patients with T2DM progressively declined by 1.2% after UC-MSCs transplantation, with a maximum decrease observed at 3 and 6 months following treatment, and slightly increased at 12-month [18]. In the present study, the HbA1c levels of the UC-MSCs group decreased 1.31% and the percentage of patients achieved the target HbA1c levels (< 7%) was the highest (55.56%) at 9 weeks and was maintained at approximately 40% during follow-up. The trend of HbA1c changes in our UC-MSCs group was similar to that published in other studies using UC-MSCs transplantation for patients with T2DM. In addition, our UC-MSCs infusion achieved a similar reduction in HbA1c levels compared with other common hypoglycemic agents to treat T2DM, which confirmed the high efficacy of UC-MSCs for the treatment of T2DM.

Animal studies showed that MSCs transplantation increased the levels of insulin and C-peptide in type 1 diabetes mellitus and T2DM by promoting the replication of residual β-cells, improving the autophagy function of islets, promoting the phenotypic dedifferentiation of islet β-cells and the polarization repair of macrophages in the local microenvironment of islets [26, 27]. In addition, some clinical studies have explored the effects of UC-MSCs on islet β-cell function of patients with T2DM. They found that fasting serum C-peptide levels progressively increased to the peak value at 6 months following UC-MSCs transplantation and then slightly decreased at 12 months, while no significant difference was observed in OGTT 2 h postprandial C-peptide levels at baseline and 12-month post-treatment [18]. Likewise, another study found no significant changes in plasma C-peptide levels at four time points (OGTT 0, 1, 2, and 3 h) after UC-MSCs infusion [19]. Consistently, no significant improvement in fasting plasma C-peptide levels in the UC-MSCs group was observed. Glucagon-stimulated C-peptide levels and AUCC-pep temporarily increased and then gradually decreased to the baseline levels after UC-MSCs infusion. Animal studies revealed that β-cell dedifferentiation rather than apoptosis was the predominant contributor to T2DM. UC-MSCs therapy was a promising strategy for reversing β-cell dedifferentiation in T2DM. Because the reversal effect of UC-MSCs was more robust at the early stages of β-cell differentiation [28], the improvement in islet β-cell function was not obvious in the patients that had the disease for a long time.

MSCs infusion has been shown to reduce insulin resistance in animal T2DM models. BM-MSCs infusion ameliorated insulin resistance of rats with T2DM by activating the insulin receptor substrate 1/protein kinase B signaling pathways [9]. UC-MSCs reduced insulin resistance in type 2 diabetic rats by eliciting adipose tissue macrophages into an anti-inflammatory phenotype [29]. In addition, adipose-derived MSCs improved hyperglycemia in type 2 diabetic rats by regulating hepatic glucose metabolism [30]. It has been reported that UC-MSCs improved insulin resistance in the liver and adipose tissues of rats with T2DM by suppressing NLRP3 inflammasome-mediated inflammation [31]. Few clinical studies have focused on the effect of UC-MSCs on insulin resistance in T2DM. In the present study, we demonstrated that UC-MSCs infusion significantly alleviated insulin resistance in a time-dependent manner, which indicated that UC-MSCs treatment was effective at improving insulin resistance in T2DM.

The therapeutic effects of UC-MSCs have been reported in a variety of diseases and no obvious adverse effects were reported [32, 33]. In our study, one patient suffered from cerebral infarction due to a long history of uncontrolled hypertension and hyperlipidemia, which was not related to UC-MSCs infusion, and no cell-related immunological reactions or tumor formation was identified. The safety assessment indicated that the administration of UC-MSCs via intravenous infusion was well tolerated in patients with T2DM. Further observations of possible transplant complications are required.

This study was a single-center trial that recruited a small number of Chinese patients. The age, course of T2DM, condition of islet β-cell function, and insulin resistance of the enrolled subjects were highly heterogeneous. Therefore, the results could not be extended to all patients with T2DM. Since our patients were followed for a short period, long-term follow-up is needed to validate the current findings. Future well-controlled studies with an increased number of cases are required to clarify the efficacy and safety of intravenous infusion of UC-MSCs for the treatment of T2DM.

## Conclusions

This prospective, single-center, randomized, double-blinded, placebo-controlled trial suggests that UC-MSCs administration via intravenous infusion is a safe and effective approach that could reduce exogenous insulin requirement and could alleviate insulin resistance in patients with T2DM. UC-MSCs transplantation could be a potential therapeutic option for T2DM.

## Supplementary Information

**Additional file 1.** Identification of human UC-MSCs: (S1A) UC-MSCs had spindle-shaped and fibroblast-like morphology. Scale bar = 100 μm; (S1B) flow cytometric analysis of cell surface markers of human UC-MSCs. The expression of each antigen was relative to the corresponding isotype control; (S1C) alizarin Red S staining of cultured osteogenic UC-MSCs. Scale bar = 100 μm; and (S1D) oil Red O staining of cultured adipogenic UC-MSCs. Scale bar = 50 μm.

## Data Availability

The datasets generated and analyzed during the present study are available from the corresponding author on reasonable request.

## Abbreviations

AUCC-pep: C-peptide area under the curve
BM-MSCs: Bone marrow-derived MSCs
CI: Confidence interval
GIR: Glucose infusion rate
HbA1c: Glycated hemoglobin
HEC: Hyperinsulinemic-euglycemic clamp
IAA: Insulin autoantibody
GIR: Glucose infusion rate
MSCs: Mesenchymal stem cells
OGTT: Oral glucose tolerance test
PLAGH: PLA General Hospital
T2DM: Type 2 diabetes mellitus
UC-MSCs: Umbilical cord-derived mesenchymal stem cells
