# Supplementary material for: Efficacy and safety of umbilical cord-derived mesenchymal stem cells in Chinese adults with type 2 diabetes: a single-center, double-blinded, randomized, placebo-controlled phase II trial
Source: Stem Cell Res Ther. 2022 May 3;13:180. doi: 10.1186/s13287-022-02848-6 (PMC9066971; doi:10.1186/s13287-022-02848-6)

Supplementary Material

**Supplementary Figure 1.** Identification of human UC-MSCs: (A) UC-MSCs had spindle-shaped and fibroblast-like morphology. Scale bar = 100 μm; (B) flow cytometric analysis of cell surface markers of human UC-MSCs. The expression of each antigen was relative to the corresponding isotype control; (C) alizarin Red S staining of cultured osteogenic UC-MSCs. Scale bar = 100 μm; and (D) oil Red O staining of cultured adipogenic UC-MSCs. Scale bar = 50 μm.


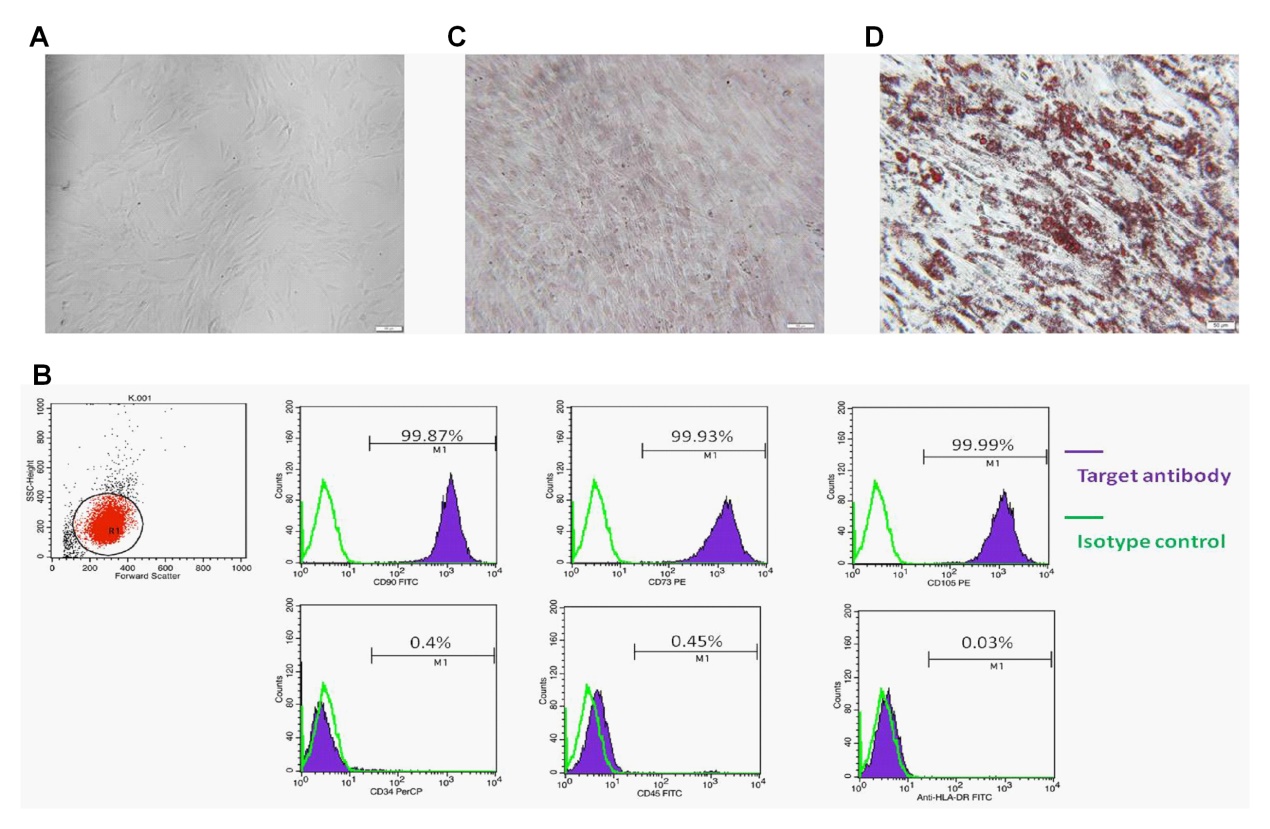

Supplement: Supplementary file 1 — Additional file 1. Identification of human UC-MSCs: (S1A) UC-MSCs had spindle-shaped and fibroblast-like morphology. Scale bar = 100 μm; (S1B) flow cytometric analysis of cell surface markers of human UC-MSCs. The expression of each antigen was relative to the corresponding isotype control; (S1C) alizarin Red S staining of cultured osteogenic UC-MSCs. Scale bar = 100 μm; and (S1D) oil Red O staining of cultured adipogenic UC-MSCs. Scale bar = 50 μm. [file 13287_2022_2848_MOESM1_ESM.docx]
